# An Evaluation of the Prescribing of High Dose Antipsychotic Therapy and Combination Antipsychotic Therapy to Inpatients on the General Adult Wards of Mersey Care NHS Foundation Trust

**DOI:** 10.1192/bjo.2022.394

**Published:** 2022-06-20

**Authors:** Louise Campbell, Harry Holmes, Declan Hyland

**Affiliations:** 1 University of Liverpool, Liverpool, United Kingdom; 2 Mersey Care NHS Foundation Trust, Liverpool, United Kingdom

## Abstract

**Aims:**

High dose antipsychotic therapy (HDAT) is defined as “a total daily dose of a single antipsychotic which exceeds the upper limit stated in the SPC or BNF or a total daily dose of two or more antipsychotics exceeding the SPC or BNF maximum using the percentage method. Previous audits have looked at HDAT on both a national level (the Prescribing Observatory for Mental Health) and within Mersey Care NHS Foundation Trust. This audit aimed to identify the proportion of patients subject to HDAT and review combination antipsychotic strategies and consideration of Clozapine in patients subject to HDAT.

**Methods:**

In August 2021, data were collected from the eight inpatient wards in Mersey Care NHS Foundation Trust. This involved using the Electronic Prescription and Administration system to identify those prescribed antipsychotics. Following this, the patient's electronic record was scrutinised for documentation of the rationale for HDAT, combination antipsychotics and consideration of Clozapine.

**Results:**

129 inpatients were identified as being prescribed antipsychotic medication. 21 (16.3%) patients were prescribed combination antipsychotic therapy, with four of these patients (3.1%) being prescribed HDAT. For these four HDAT patients, there was no recorded documentation of discussion of the option of Clozapine. The most common antipsychotic combination was Paliperidone depot with oral Risperidone. 38 out of 129 (29.5%) patients had been considered for Clozapine. Reasons for Clozapine being refused included the patient declining, concerns about non-concordance with oral medication, patients having had a neutropenia on an FBC, the patient being reluctant to have regular blood tests and a patient's comorbidities.

**Conclusion:**

When comparing the proportion of patients subject to HDAT (3.1%) to the previous Trust audit in December 2020 (9.1%), there is a recurrent theme that antipsychotic prescribing practice in Mersey Care is safe, with minimal HDAT. Of note, the figure is significantly lower than the proportion of HDAT patients identified in the 2012 national study (28%). In this audit, none of the patients on HDAT had documented consideration of Clozapine. Three of the four patients were soon to be no longer subject to HDAT which may explain this result. Compared to the Trust's HDAT audit in 2020, the percentage of patients on combination antipsychotic therapy has stayed largely the same - 16.3% compared to 17.4%. The Trust needs to strive to continue minimal HDAT prescriptions and ensure that, in those patients subject to HDAT, there is consideration of and documentation of Clozapine being considered.

